# Rescue Re-Do Inline Osteosynthesis with Wire Cerclage for Failed Rib Plating of Multilevel Rib Nonunion

**DOI:** 10.4103/jctt.jctt_13_19

**Published:** 2019-12-30

**Authors:** Tatiana Kazakova, Marcel Tafen, Warner Wang, Roman Petrov

**Affiliations:** 1 Department of Family Medicine, Jefferson Health; 2 Department of Surgery, Albany Medical College, Albany, NY; 3 Department of Surgery, Marietta Memorial Hospital, Marietta, Ohio, USA; 4 Department of Thoracic Medicine and Surgery, Temple University Hospital, Philadelphia, PA

**Keywords:** Hardware failure, redo rib repair, rib cerclage, rib fractures, rib nonunion, rib osteosynthesis, rib plating

## Abstract

Rib nonunion is a rare occurrence that requires surgical management and has a high rate of failure that may necessitate repeated intervention. We present the case of successful rescue redo repair of previously failed plating of chronic nonunion for multilevel posterior rib fractures, reinforced by wire cerclage of the osteosynthesis plate. Our objective is to illustrate the feasibility of repeated interventions, and the technique to resolve this challenging problem.

## Introduction

Rib fractures occur in about 10% of trauma admission.^[1]^ Mainstay management is supportive care and pain control. With this approach, most rib fractures will heal; however, a small subset will not heal.^[2]^ Surgical stabilization with inline osteosynthesis for nonunion of rib fractures is a valuable option for the restoration of function and addressing the associated chronic pain syndrome. However, posterior rib fractures at the angle of rib are a specifically challenging problem due to difficult exposure, contour angulation, and shortened proximal rib segment anchoring for the adequate plate purchase, limited by the transverse process. We present a case of successful rescue redo repair of previously failed plating of chronic nonunion for multilevel posterior rib fractures, reinforced by wire cerclage of the osteosynthesis plate.

## Case Report

**Figure 1:**
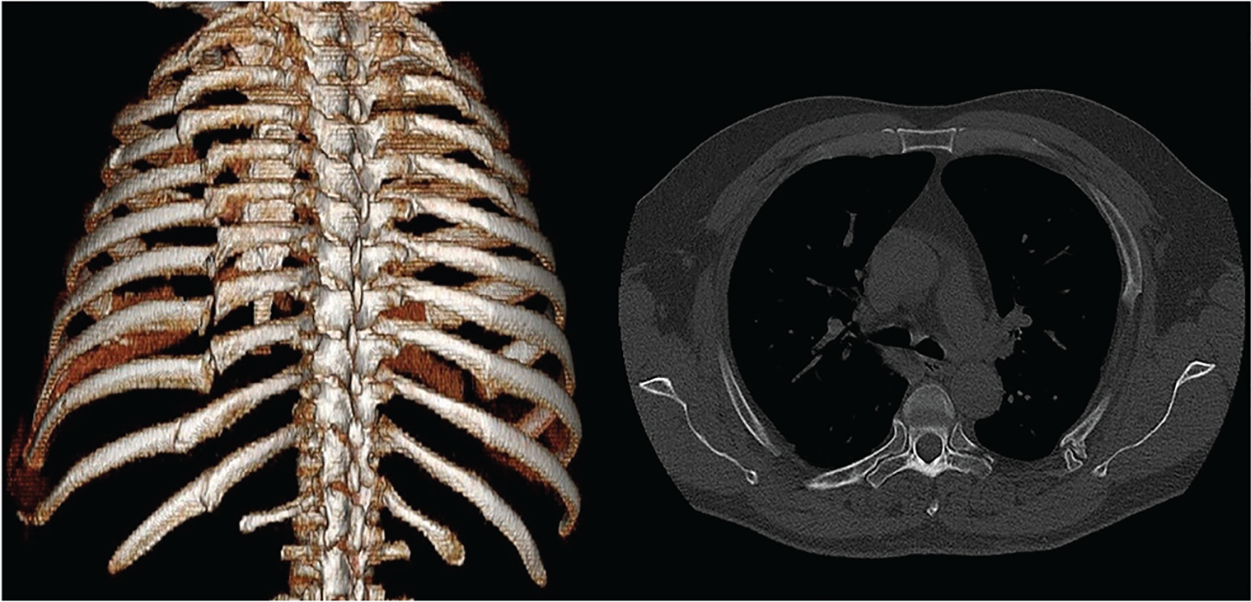
Computed tomography demonstrating multiple healed posterior rib fractures of ribs 2 through 12 with chronic rib fracture nonunion of ribs 6 through 10

**Figure 2:**
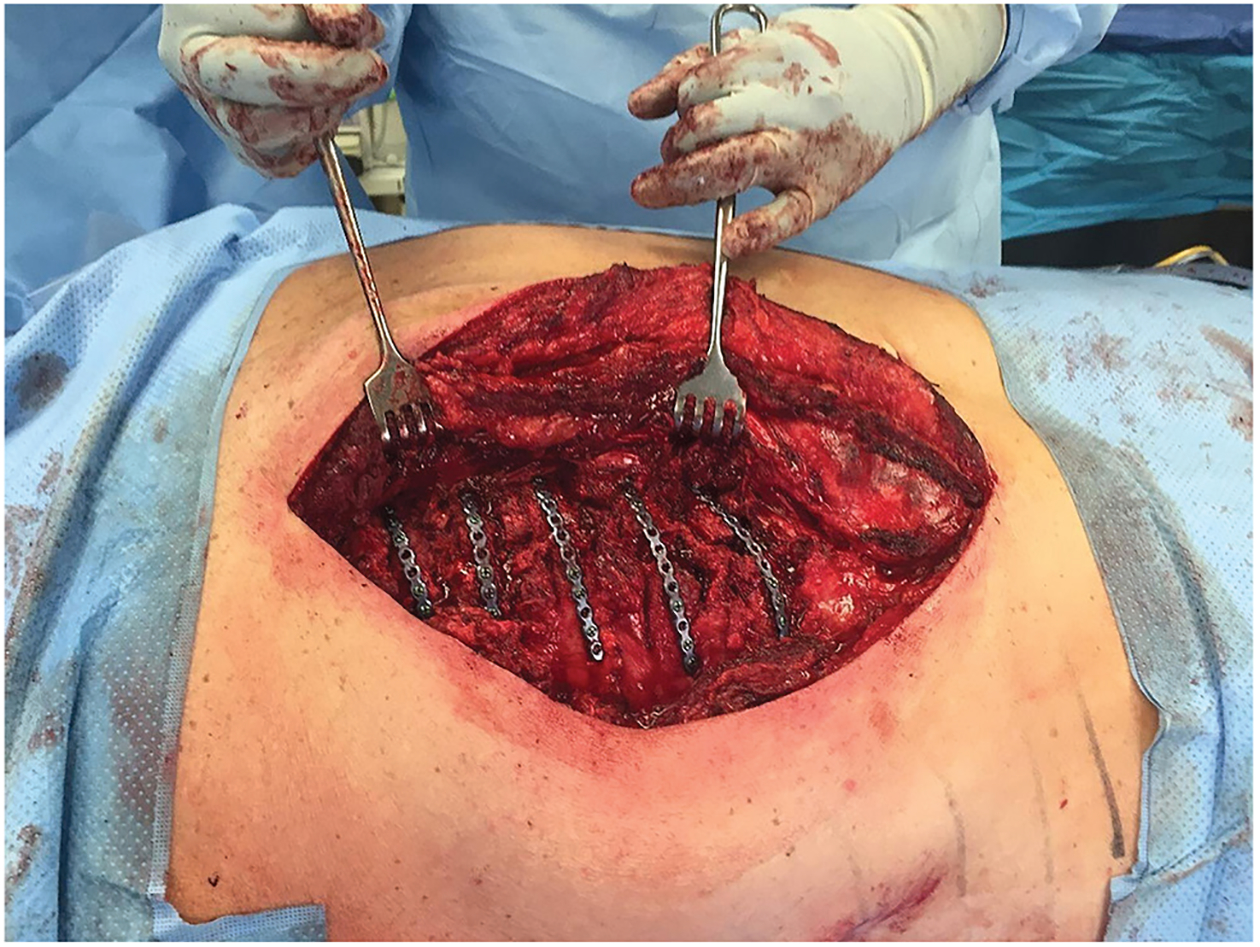
Intraoperative image of the original in-line osteosynthesis of ribs 6–10. Patient is in prone position with left paraspinal incision with head toward left of the picture

**Figure 3:**
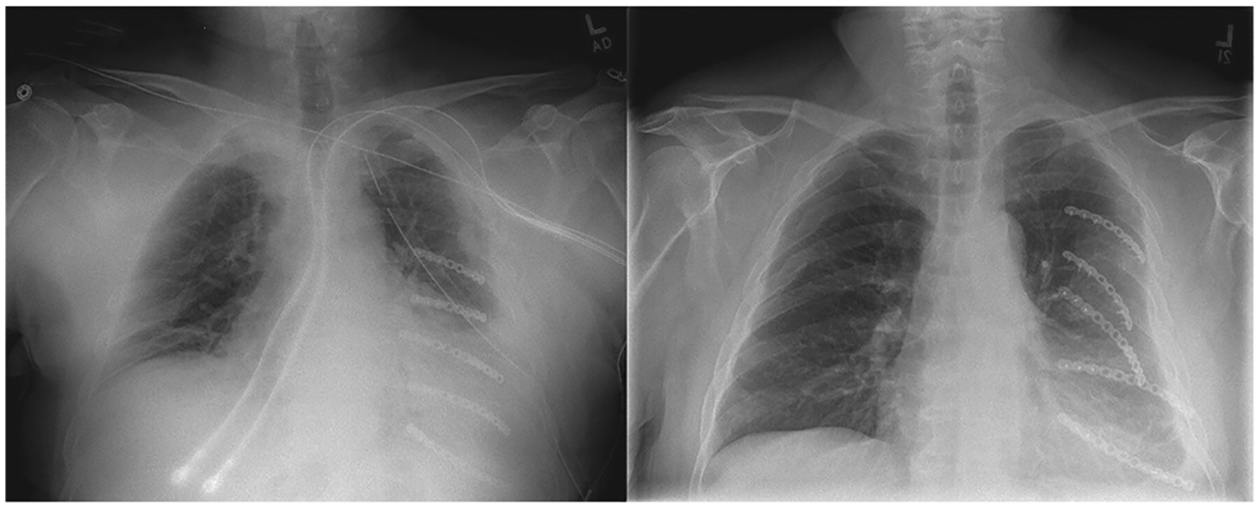
Immediate postoperative and first clinic follow-up chest X-ray demonstrating progressive hardware failure with displacement of the plates

**Figure 4:**
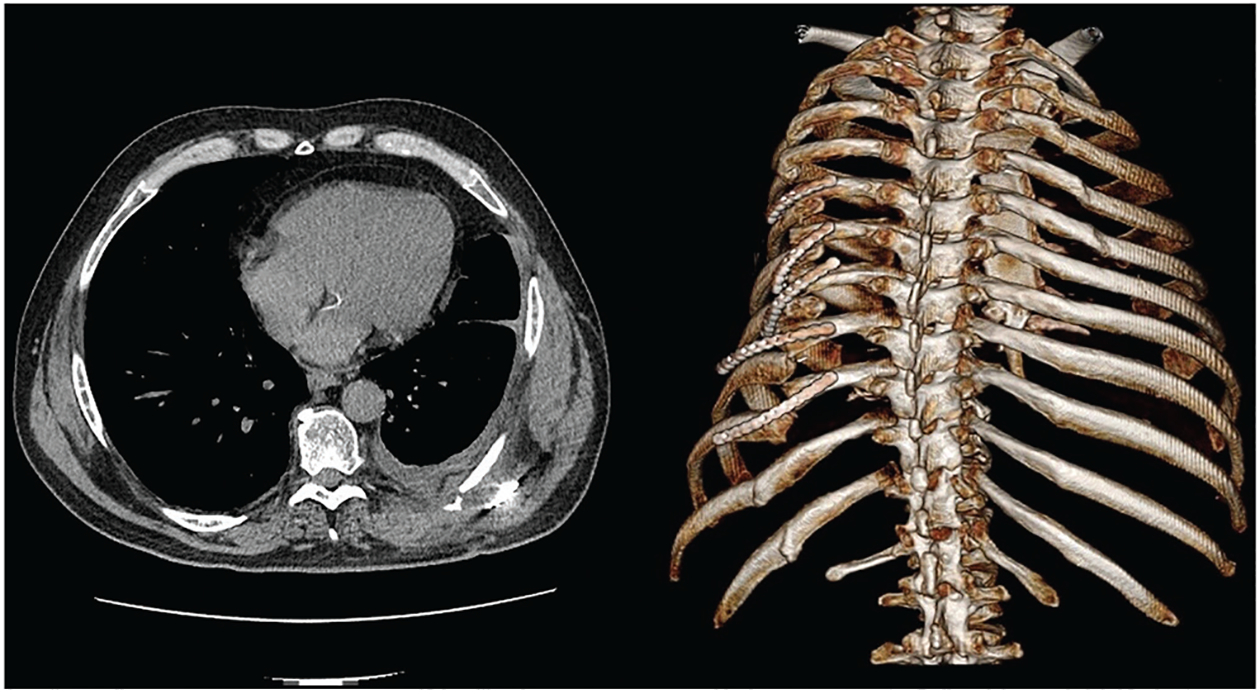
Computed tomography demonstrating hardware failure with pull out of the screws from rib stump and chest wall collapse

**Figure 5:**
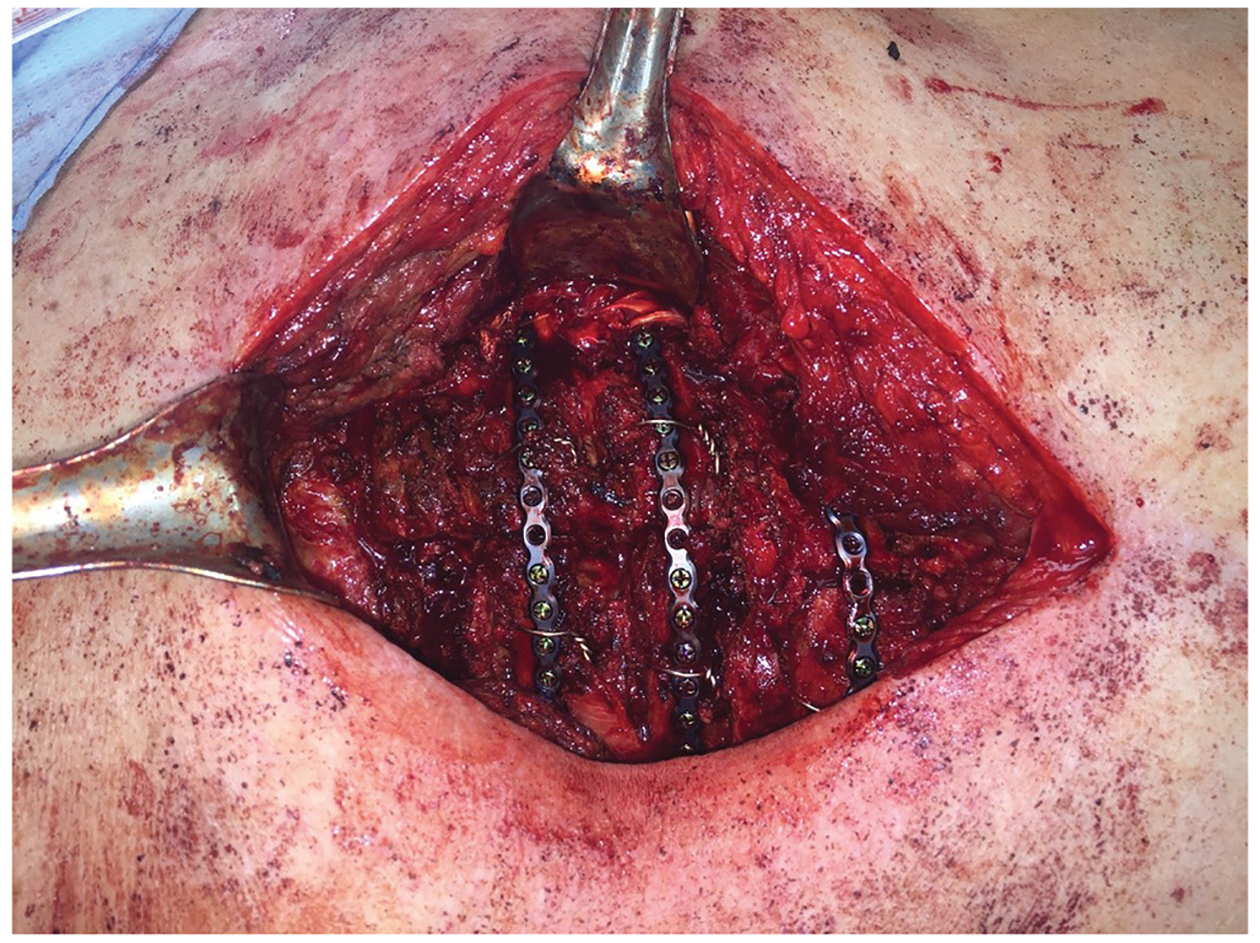
Intraoperative image of rescue in line rib osteosynthesis with wire cerclage of ribs 6-through 9

**Figure 6:**
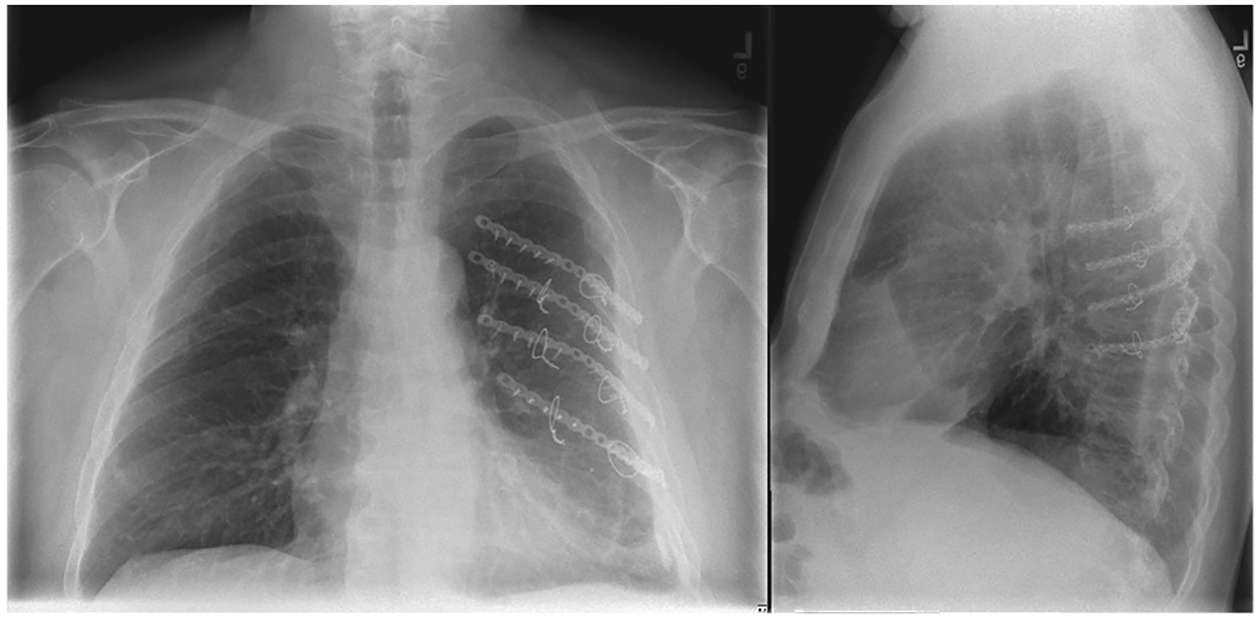
Postoperative imaging of the rescue repair after lower plate explanation 6 months after rescue repair. Hardware remains competent

S. N. is a 64-year-old male, physically active, construction worker, with excellent performance status who presented with severe chronic pain, after sustaining multiple left rib fractures due to blunt trauma 16 months prior. The patient was referred to by pain management specialist after failing medical and interventional therapy. Workup revealed healed multiple left rib fractures with chronic nonunion with “elephant leg” deformity at the angle of the rib of the left 6^th^ through 10^th^ ribs [Figure 1]. The patient underwent resection of the nonunions and inline osteosynthesis of ribs 6 through 10. We used locking RibFix Blu system (Zimmer Biomet, Warsaw, IN) plates with bicortical screw fixation. Care was taken to place three screws on each side of the fracture without immediate complications [Figure 2]. With early postoperatively, the patient continued to complain of recurrent pain. Repeat evaluation revealed the failure of the hardware with pull out of the screws from ribs fragment and collapse of the chest wall [Figures 3 and 4]. Decision was made to proceed with rescue repair 8 weeks after the original procedure. This time, longer plates were selected, anchoring with at least five screws on each side of the fracture and reinforcing this construct with #7 metal wire cerclage of ribs 6 through 9 [Figures 5 and 6]. The tenth rib plate appeared competent and was left intact. After an uneventful recovery, the patient reported a significant improvement of pain. Several months later, the patient reported the recurrence of pain in the projection of the lower plate. Physical examination demonstrated piano key sign and imaging confirmed similar mode of hardware failure with pullout of screws from proximal stump of the rib. Six months after second intervention the patient was reexplored for the third time where failed hardware with complete union of the tenth rib fracture was found. This plate was explanted with complete resolution of pain. The patient remains asymptomatic at 2 years follow-up.

## Discussion

Traumatic rib fractures are common in blunt trauma and frequently are managed conservatively. An emerging body of literature demonstrates the benefits of surgical repair of acute rib fractures.^[3]^ Surgical stabilization of ribs has been shown to shorten length of stay reduce rates of respiratory failure, pneumonia, intensive care unit stay, and mortality.^[4–9]^ Only 1% of patients with rib fractures undergo surgical stabilization, with a vast majority being treated non-operatively.^[10]^ Rib fracture patients often do not return to work, develop chronic pain, dyspnea and in a small portion of patients nonunion develops.^[11–13]^ Nonunion of ribs mainly manifests with chronic pain, and traditional pain management modalities are rarely effective.^[14]^ Limited data suggest positive outcomes with surgical repair of rib nonunion.^[15,16]^

However, surgical management of the nonunion represents significant challenges. Bone contour distortion frequently demands resection of the site of nonunion for realignment. Sufficient immobilization of the fracture is subsequently required to facilitate an altered healing process.^[14,15,17]^

Notably, posterior rib fractures, especially the angle of the rib, are particularly challenging due to a short proximal rib fragment and contour angulation.^[18]^ The short posterior segment and the proximity to the transverse vertebral process restrict the proximal landing zone and purchase of the osteosynthesis plate.^[19]^ The acute angulation renders it specifically difficult contouring the plate for satisfactory alignment. In addition, the long anterior segment of the rib serves as a lever, exerting excessive torque on the area of fracture, leading to displacement, and hardware failure. Potentially, “U” plates like the RibLock U + system (Acute Innovations, Hillsboro, OR) can be advantageous due to the circumferential fixation. However, in the case of angle of rib fracture it is difficult to align “U”-plate with the rib. Our technical solution with wire cerclage in addition to longer segment with at least five screws proved sufficient.

One concern exists about the galvanic pair of the titanium plate and stainless-steel wire. However, in the experimental settings, this combination did not produce adverse outcomes.^[20]^ We have not observed any adverse events of addition of the wire cerclage to the repair during the 2 years of follow-up. Alternatively, Sternal ZIPFIX® System (Synthes, West Chester, PA, USA) could be used for the same purpose. We feel that this was the deciding factor to the successful resolution of this difficult problem.

To the best of our knowledge, this is the first case of rescue osteosynthesis of posterior rib nonunion repair with the use of wire cerclage in the literature. This case demonstrates the feasibility of repeated repair for the management of chronic posttraumatic pain caused by rib fracture nonunion and the technical solution for this anatomically difficult problem.
